# Unusual Localization of a Primary Hydatid Cyst: Scaphoid Bone: A Case Report

**DOI:** 10.1097/MD.0000000000003290

**Published:** 2016-04-29

**Authors:** Sancar Serbest, Ugur Tiftikci, Abuzer Uludag

**Affiliations:** From the Faculty of Medicine, Department of Orthopedics and Traumatology, Kırıkkale University, Kırıkkale (SS, UT) and Faculty of Medicine, Department of Orthopedics and Traumatology, Adıyaman University, Adıyaman (AU), Turkey.

## Abstract

Because hydatidosis of the bone (echinococcus infection) is a rare parasitic infection, its diagnosis and treatment poses great difficulties. Radiologic imaging findings are generally helpful to make the diagnosis. But occurrence of disease in atypical places and lack of specific radiological findings may complicate differential diagnosis. Nevertheless, familiarity with imaging findings in patients living at endemic areas provides advantages for diagnosis and treatment.

We present a cyst hydatic case in scaphoid bone which has been reported in the literature only once previously.

## INTRODUCTION

Cystic echinococcus or hydatidosis is a parasitic infection of humans and animals. This parasite has 12 different types although 4 types cause disease in humans: *Echinococcus granulosus*, *Echinococcus multilocularis*, *Echinococcus vogeli*, and *Echinococcus oligarthrus*.^1^ Hydatic cyst occurs in many areas of the world but it is especially endemic in South America, Asia, Australia, North and East Africa, and Middle East where sheep and cattle breeding is a common occupation.^2^ Incidence of hydatic cyst disease ranges between 3 and 50 per 100,000 population.^3,4^

Although hydatic cyst can form disease in any organ except hair and nails, it usually affects liver, spleen, and lungs.^5^ Involvement of these 3 organs is responsible from 90% of infections.^6^ Primarily bone involvement of cyst hydatic occurs in 1% to 2.4% and among them 30% to 50% involves the vertebrae, 15% involves pelvis, and less frequently involves long bones.^3,7^

## CASE PRESENTATION

**FIGURE 1:**
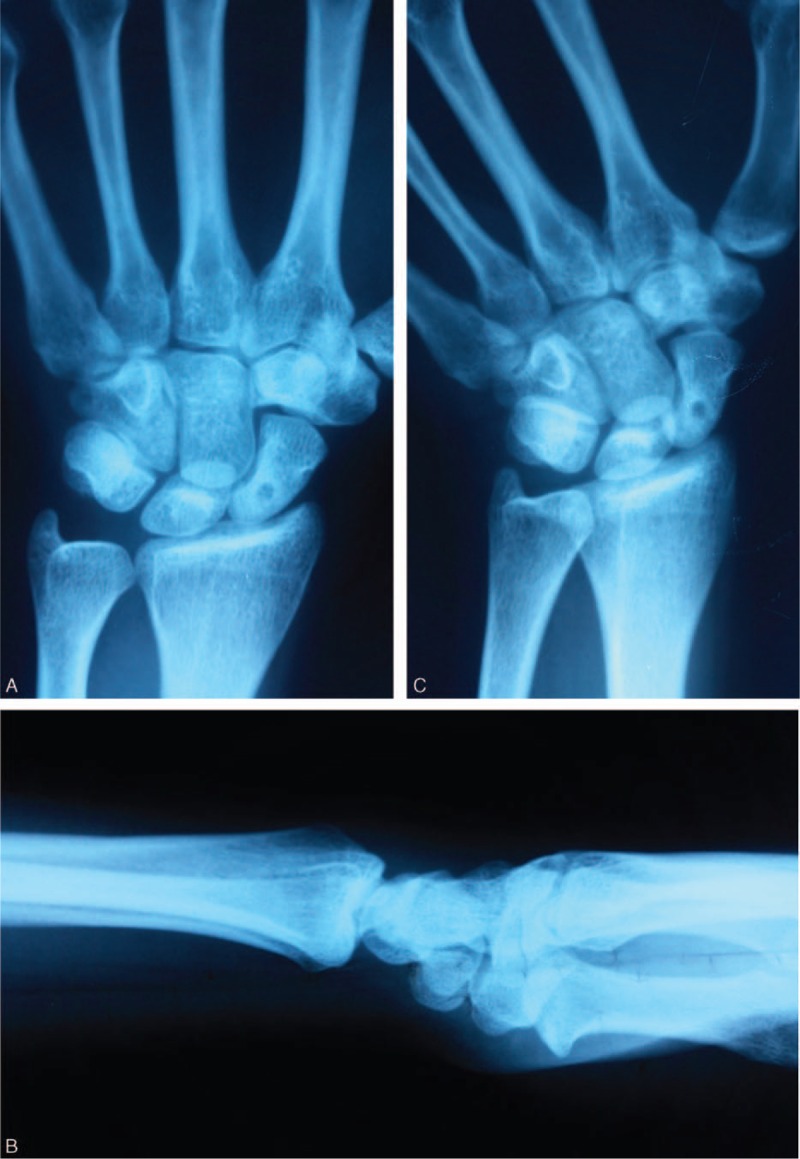
Anteroposterior (A), lateral (B), and scaphoid (C) X-rays of the patient. In these figures a well demarcated cyst is seen at proximal pole of scaphoid.

**FIGURE 2:**
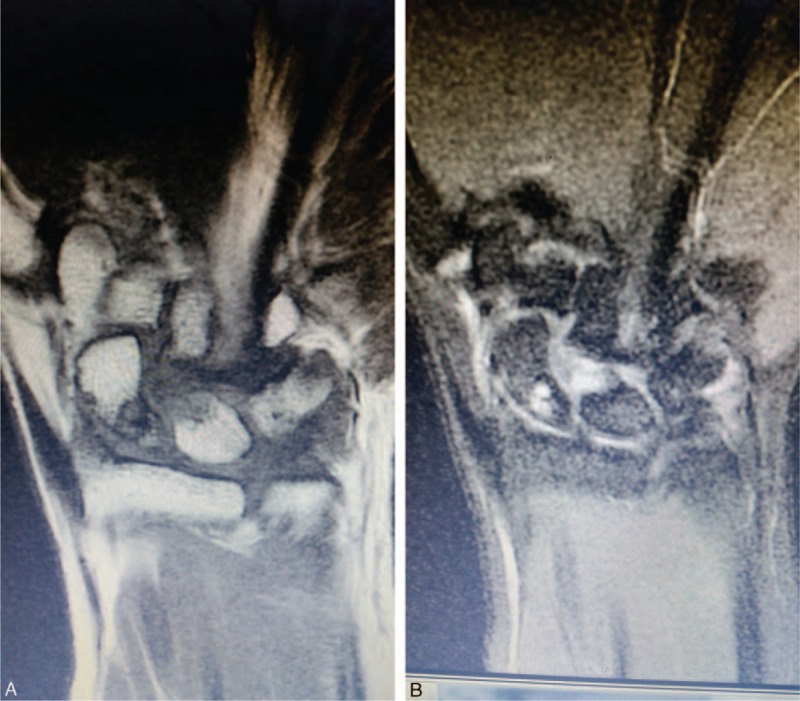
T1 (A) and T2 (B) weighted coronal cross section of the preoperative magnetic resonance imaging shows a bone cyst of 0.5 cm at proximal pole of scaphoid.

**FIGURE 3:**
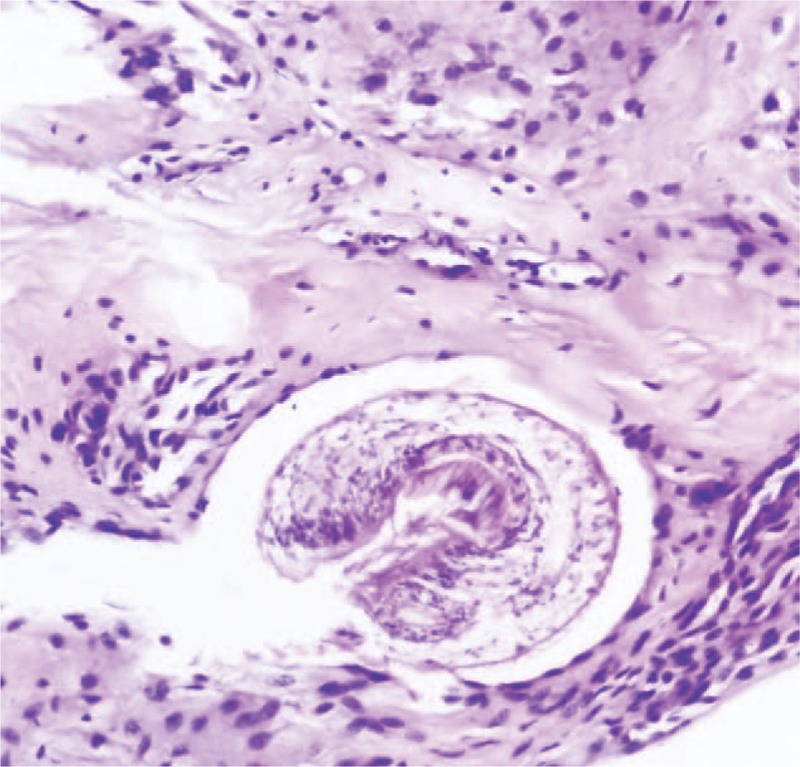
Scolex is seen inside cystic space in fibrous tissue infiltrated by inflammatory cells (Hematoxylin eosin stain; magnification ×200).

**FIGURE 4:**
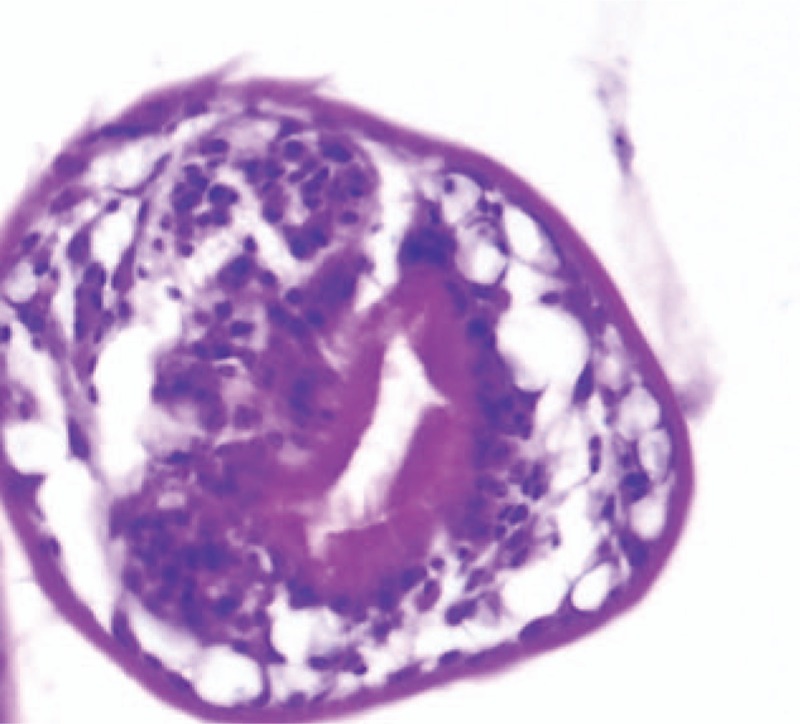
High power magnification image of scolex (Hematoxylin eosin stain; magnification ×400).

A 22-year-old female patient came to our clinic with a complaint of pain in her left wrist. Her clinical history revealed that she fell on this wrist approximately 2 months before and the pain started afterwards. Her physical examination showed mildly painful wrist movements with minimal limitation. Anatomically there were mild tenderness and swelling in fossa radialis. There was no other systemic complaint. Her personal and family histories were unrevealing. Wrist and scaphoid X-rays were performed. The radiographic examination revealed a well demarcated cystic structure at proximal pole of the scaphoid (Figure 1). Magnetic resonance imaging confirmed a 0.5-cm diameter bony cyst at proximal pole of the scaphoid (Figure 2). Laboratory evaluations including complete blood count, C-reactive protein, erythrocyte sedimentation rate, and urine analysis were within normal limits except eosinophilia. Cyst hydatic indirect hemagglutination (IHA) test and indirect immunofluorescence antibody test were reported to be negative. We planned surgical treatment with an initial diagnosis of wrist pain due to simple bone cyst. After a tourniquet was applied to forearm, scaphoid was reached by volar approach. A light colored liquid and a white membrane resembling germinative membrane of hydatic cyst were observed. Curettage was applied to cystic area and it was flashed with abundant hypertonic solution and povidone iodine solution. No mechanical bone damage was seen. After curettage the formed cavity was filled with spongious allograft. Two weeks after the surgery short-arm splint with thumb support was applied. Histopathological evaluation revealed cyst hydatic scolexes in cystic spaces inside fibrous tissue and inflammatory cellular infiltrates inside surrounding fibrous tissue (Figures 3 and 4).

The patient was consulted by an infectious diseases specialist and a general surgeon. To exclude involvement of another organ, chest X-ray and abdominal ultrasonography were ordered. No cystic mass was observed in thorax or abdomen. The patient took daily 2 × 400 mg albendazol oral for 3 months. After 2 months, the splint was removed and wrist exercises were started. Two months after the surgery, swelling in fossa radialis terminated. Wrist movements were painless and complete. The patient was evaluated by clinical examination, serological tests, and radiological imaging at 3rd, 6th, and 12th month follow-up visits. No finding related with local or systemic hydatic cyst was detected at 12th month follow-up. Recurrence was not observed at 12th month follow-up visit and serological tests were negative.

## DISCUSSION

Hydatic cyst has a main and an intermediate host and it is transmitted to humans by direct contact or consumption of contaminated food. After ingestion, Echinococcus eggs reach portal venous circulation after passing through duodenal mucosa and cause disease after arriving to liver and the lung. Liver and lung function as filters for the parasite. On the other hand, some eggs may localize to peripheral organs after passing from lung. The parasite is transformed to larval stage in which it develops hydatic cyst in the end organ its egg stays.^8^

Involvement of many bones and soft tissues other than liver, lung, or spleen have been reported previously.^9–14^ Although hydatic cyst in bones may form due to hydatic cyst of liver or lung, it generally occurs as primary bone involvement in the absence of liver or lung involvement.^15,16^ Involvement of carpal bones is rare and only 1 case has been reported in proximal pole of scaphoid bone.^9^ Interestingly although size of the lesion was different in that case its localization was same with ours.

Differential diagnosis of a cystic lesion in carpal bones includes infections due to typical and atypical agents (including tuberculosis, brucellosis, and parasitic infections), fibrous dysplasia, simple bone cysts, osteosarcoma, intraosseous gangliomas, or osteomyelitis.^17,18^ Osseous hydatic is the name given to unilocular cysts in a bone. As seen in our patient, these patients usually present with complaints of pain and swelling. Deformity, pathological fractures, and secondary infections are also among symptoms.^19,20^

Diagnosis of cyst hydatic is difficult due to lack of a biochemical or radiological finding specific to cyst hydatic. Definite diagnosis can be established by histopathological examination of cyst material. Although sclerosis or periost reaction is not prominent at early stages of the disease, lytic lesions accompanied by sclerosis may occur at later stages. Our case also had a lytic lesion accompanied by sclerosis. Although computed tomography gives additional information regarding localization and calcification of the cyst, magnetic resonance imaging gives information about the severity of bone and soft tissue involvement.^5,18^

Eosinophilia is an important laboratory finding; however, it is elevated only in 25% of all cases. Additionally, positivity of IHA test is also important in hydatic cyst diagnosis but negative IHA test result does not exclude the diagnosis.^21,22^ In our case, IHA test was negative although eosinophilia was present.

There is not an accepted treatment protocol for cyst hydatic localized to bone. This is mainly due to inadequate number of cases.^9^ Early diagnosis and treatment is important to prevent complications. Main principal of the treatment is abundant irrigation of cyst pouch^22^ with a scolocidal agent such as 5% silver nitrate,^23^ hypertonic saline,^21^ or povidone iodine solution after excision and curettage. Formed bone defects may be filled with polymethylmetacrylate,^11^ autograft, or allograft.^21^ To decrease recurrence rate antihelminthic treatment should be given both preoperatively and postoperatively. Recommended treatment course is approximately 3 months.^24,25^

In conclusion, primary cyst hydatic in scaphoid bone is very rare. Simple cystic structures in carpal bones are frequent and in patients with clinical symptoms, especially if they live in an endemic region for *E granulosus*, hydatic cyst should be considered in the differential diagnosis of these cystic structures.
